# Dynamic postural stability and asymmetry in thigh circumference and single‐leg hop test following anterior cruciate ligament reconstruction

**DOI:** 10.1002/jeo2.70353

**Published:** 2025-07-13

**Authors:** Aguri Kamitani, Derrick M. Knapik, Matthew V. Smith, John Motley, Sina Tartibi, Amanda Haas, Rick Wright, Matthew J. Matava, Robert H. Brophy

**Affiliations:** ^1^ Department of Orthopaedic Surgery Washington University St. Louis Missouri USA; ^2^ Department of Orthopaedics Graduate School of Medical Science Kyoto Prefectural University of Medicine Kyoto Japan; ^3^ STAR Physical Therapy Barnes‐Jewish Hospital St. Louis Missouri USA

**Keywords:** ACLR, dynamic postural stability, knee, limb symmetry index, single‐leg hop

## Abstract

**Purpose:**

To evaluate changes in limb symmetry index (LSI) in thigh circumference and single‐leg hop for distance (SLHD) after anterior cruciate ligament reconstruction (ACLR) out to 18 months postoperatively and their association to changes in dynamic postural stability after ACLR out to 24 months.

**Methods:**

Patients were prospectively enroled after ACLR and followed up at 3‐month intervals. Thigh circumference was measured preoperatively out to 18 months postoperatively, dynamic postural stability out to 24 months, and SLHD from 6 to 18 months following ACLR. LSI was calculated from the thigh circumference and SLHD measurements. Dynamic postural stability (DPS) was measured on a multidirectional platform that tracked the patient's centre of mass, creating a dynamic motion analysis (DMA) score that reflected ability to maintain their centre of mass.

**Results:**

A total of 47 patients with mean age of 19.1 ± 5.8 years completed the study. LSI in thigh circumference worsened initially and improved at longer follow‐up. LSI in SLHD improved significantly at 9‐ and 12‐months consecutively. Overall mean DMA scores improved significantly at 3‐ and 6‐month postoperatively. No significant correlation between LSI and DMA scores was appreciated at any time point.

**Conclusions:**

LSI in thigh circumference decreases initially after ACLR and then improves, while LSI in SLHD and dynamic postural stability improved after ACLR. DPS improved primarily in translational planes of motion. No significant association between LSI in thigh circumference/SLHD and DPS was found at any follow‐up point.

**Level of Evidence:**

Level IV, case series.

**Abbreviations**
ACL: anterior cruciate ligament
ACLR: anterior cruciate ligament reconstruction
BMI: body mass index
BPTB: bone–patellar‐tendon–bone
cm: centimetre
DMA: dynamic motion analysis
DPS: dynamic postural stability
HT: hamstring tendon
kg: kilogram
LSI: limb symmetry index
m: metre
MRI: magnetic resonance imaging
NR: not reported
RTS: return to sport
SD: standard deviation
SLHD: single‐leg hop for distance

## INTRODUCTION

Injuries to the anterior cruciate ligament (ACL) represent one of the most common and devastating sports injuries in active adolescents and athletes. ACL reconstruction (ACLR) is recommended to restore stability to the knee and minimise the risk for further injury to facilitate return to sport (RTS) and preinjury activities in active patients [5, 6]. However, successful RTS rates following ACL injury and subsequent reconstruction have been reported to range from 55% to 81% [3, 12], with a high risk for recurrent ACL injury, especially during the first 2 years following surgery [34]. Specifically, active athletes returning to sports possess an approximately 30–40x greater risk for recurrent ACL injury when compared to uninjured counterparts [45]. One of the most commonly cited factors hindering successful RTS and subsequent risk for ACL graft retear is the loss of proprioception following index ACL injury [9, 37]. While the implementation of appropriate rehabilitation regimens incorporating exercises to help regain neuromuscular control and postural stability following ACLR have been well documented [8, 26], patient‐specific factors influencing the time to regain postural stability and the extent to which it is regained remains largely unknown.

Asymmetries in lower limb strength and kinematics during landing tasks have been cited to impede successful RTS following surgery, with persistent deficits increasing the risk for secondary injuries after RTS [19, 21, 22, 36]. The limb symmetry index (LSI) is the degree to which the single‐leg hop for distance (SLHD) is reestablished. It represents an objective measure of knee function which is commonly utilised when determining the appropriate time to RTS [42]. The achievement of >90% LSI during functional hop tests has traditionally been cited as a necessary threshold to allow RTS [44]. Meanwhile, some literatures used thigh circumference for postoperative evaluation in knee surgery of the quadriceps on the involved leg [40]. Ross and Worrell investigated the postoperative thigh circumference difference comparing with uninvolved side and described that it decreased immediately after surgery, concluded that a rehabilitation approach to reduce the difference in thigh circumference is important [40]. Allison et al. and Arangio et al. showed that thigh girth asymmetry is significantly related to knee extension and flexion torque asymmetry [1, 2].

However, both the SLHD and thigh circumference measurement fail to detect subtle limb asymmetries, perhaps contributing to continued deficiencies in postural control and proprioception following ACLR [23]. As a result, measurement of dynamic postural stability in multiple movement planes has been investigated to quantify restoration of lower extremity neuromuscular control following ACL reconstruction [8, 26]. Dynamic postural stability has been shown to incrementally improve over 12 months following ACLR, with deficits in proprioception persisting up to 2 years [8, 13]. We are not aware of any studies with 2‐year follow‐up of LSI including thigh circumference and SLHD after ACL reconstruction as serial measurements have been reported, although there are a few reports describing changes in SLHD over time within 1 year [16, 28, 32]. Furthermore, the association between changes in dynamic postural stability, as measured by asymmetries in SLHD and thigh circumference, following ACLR has not been investigated.

As such, a better understanding of the relationship between patient‐specific factors influencing restoration of dynamic postural stability during rehabilitation following ACLR may help clinicians identify and correct modifiable risk factors to minimise the risk of secondary injury. The purpose of this study was to investigate the trends in LSI based on measurements of thigh circumference and SLHD preoperatively and at consecutive 3‐months intervals up to 18 months and their association to changes in dynamic postural stability up to 2 years following ACLR. Our hypotheses were twofold: (1) that the LSI of thigh circumference and SLHD would improve significantly during the first postoperative year, followed by gradual improvement until the 2‐year follow‐up, and (2) that there would be a positive correlation between thigh circumference/SLHD symmetry and dynamic postural stability during the study period.

## METHODS

### Patients

Skeletally‐mature patients with complete ACL rupture confirmed on magnetic resonance imaging (MRI) who were scheduled to undergo primary ACLR, were eligible for study inclusion. Skeletal maturity was determined by the presence of closed distal femoral and proximal tibial physes on standard knee radiographs. Eligible patients were prospectively enroled and underwent ACLR by one of three fellowship‐trained surgeons using either bone‐patellar tendon‐bone (BPTB) autograft or hamstrings tendon (HT) autograft, over a 41‐month period. Postoperatively, the patients participated in a graduated, standardised rehabilitation protocol, concentrating on controlling swelling and regaining full range of motion, followed by incorporation of closed‐kinetic chain exercises to regain strength, coordination, and neuromuscular balance. Progression to agility, plyometric, running and sport‐specific activities, were allowed based on time from surgery and the achievement of various therapeutic milestones.

Patients were excluded if they possessed one or more of the following variables: (1) a history of knee injury or surgery prior to ACLR, (2) the presence of concurrent chondral or ligamentous (posterior cruciate ligament, medial collateral ligament) pathology requiring treatment during ACLR, (3) contralateral knee pathology or lower extremity neurologic or physiologic deficits preventing performance of postoperative rehabilitation, or (4) the presence of any other general condition affecting proprioception and postural stability. Any patients lost to follow‐up or missing two consecutive follow‐up appointments, were similarly excluded. The treatment of concomitant meniscal tears at the time of ACLR was not a contraindication for study inclusion. Patients underwent meniscal repair versus meniscectomy based on optimal surgical management and various patient and injury specific factors, favouring repair in younger patients, when clinically indicated.

Patient age, sex, height and weight at the time of preoperative evaluation were recorded and body mass index (BMI, weight (kg)/[height (m)]^2^) was calculated. The Marx Activity Score, a maximum 16‐point scale that evaluates the frequency of running, cutting, deceleration, and pivoting during the 12 months prior to injury, was also collected prior to ACLR [29].

### Thigh circumference measurement

All eligible patients underwent preoperative thigh circumference measurements by a single orthopaedic surgeon [RHB]. Measurements were performed twice on both the operative and non‐operative thigh at 10 cm and 20 cm proximal to the superior patella pole using a standard tape measure with the patients in a supine position [40]. Mean thigh circumference was calculated by averaging the two measurements at each position. Subsequent measurements were performed every 3 months following surgery, up to 18 months postoperatively. The ratio of the operative limb measurement divided by the non‐operative limb measurement was used to calculate the LSI (expressed as a percentage) at each time point.

### Single‐leg hop for distance

Beginning at 6 months following ACLR, all patients were assessed for SLHD on both the operative and non‐operative leg, and then at every 3‐month interval up to 18 months. All patients were instructed to hop forward as far as they could with their arms on their hips after standing and landing on the tested limb. A total of three hops on each limb, in which a successful landing was achieved, were required to complete the test. If a patient failed to maintain their balance while landing on either limb, they were allowed to repeat the test until completing three successful landings. Total jump distance was measured using a tape measure calibrated from the toe in the starting position to the heel in the landing position [32, 46]. The mean distance for all three hop tests was calculated, as well as the LSI between extremities.

### Dynamic postural stability testing

All eligible patients underwent dynamic postural stability testing utilising a motorised multidirectional platform (PROPRIO 5000 Reactive Balance System: Perry Dynamics, Decatur, IL) as previously described [8, 41] at a minimum of 2 days prior to surgery. The PROPRIO platform quantifies user balance and reaction ability by exerting simultaneous motion in the anterior/posterior and medial/lateral planes to measure dynamic motion analysis (DMA), reflective of a user's centre of mass when reacting to the dynamic stimulus [7, 26, 35]. The patient stands on both feet, shoulder width apart, with the knees slightly flexed. DMA testing was subsequently performed at every 3‐month interval, beginning at 3 months postoperatively up to 18 months, with final testing performed at the 24‐month follow‐up.

A total of three 2‐min DMA test trials were performed on all patients by a single author [JM]. Through use of integrated software, the overall mean DMA (ranging from 0 to 1440 points) from the three trials was calculated. DMA scores were then calculated in the six independent planes of motion, representing alterations in the patient's movement of the centre of mass away from the starting position. These included three translational plane assessments (medial/lateral, anterior/posterior and up/down) and three rotational plane assessments of the pelvis (left/right, flexion/extension and internal/external rotation). Medial/lateral translation was defined as movement measured at the position sensor when weight shifted from one lower extremity to the other. Anterior/posterior translation was defined as motion resulting from a shift of weight to the toes or heels. Up/down translation was defined as compression or distraction from the starting position, with patients typically squatting lower to control for larger perturbations and standing up or extending when fatigued. Left/right rotation was defined as the degree of pelvic tilt to the left or right. Flexion/extension rotation was defined as motion of the pelvis in the sagittal plane, and internal/external rotation was measured by the overall pelvic displacement in the transverse plane [45]. Lower scores were reflective of less displacement, consistent with improved dynamic postural stability. The mean total time in which the patient was able to maintain both feet on the platform among the three trials at each time point was also recorded.

### Statistical analysis

For each testing trial, the mean values of the three trials were calculated for the overall DMA, as well as mean DMA scores in each plane of motion and the mean length of time patients remained on the platform. Repeated measures analysis of variance with Bonferroni post hoc testing was used to compare differences in the LSI for thigh circumferences, SLHD, overall and individual DMA scores, and time on the platform, between each time point. Spearman's correlation test was used to examine for any correlation between LSI for mean thigh circumference and SLHD values on DMA scores. An unpaired *t*‐test for normally distributed cases and a Mann‐Whitney U test for not normally distributed cases were used to assess the influence of sex differences, compared clinical variables (LSI of femoral circumference, SLHD, overall and individual DMA scores, and time on platform) by sex (female vs. male). Repeated measures analysis of variance with Bonferroni post hoc test was used to compare differences in the clinical variables by presence or absence of meniscal treatment (N: no meniscal treatments vs. M: meniscectomy vs. R: meniscal repair). Statistical significance was set at a *p*‐value < 0.05. All statistical analyses were performed with EZR25 (Saitama Medical Centre, Jichi Medical University, Saitama, Japan), which is a graphical user interface for R (The R Foundation for Statistical Computing, Vienna, Austria). More precisely, it is a modified version of R commander designed to add statistical functions frequently used in biostatistics [20]. A post hoc power analysis was performed (*α* value, 0.05; *β* value, 0.8) with respect to LSI for SLHD to determine minimum required sample size.

## RESULTS

### Patient population

Out of a total of 69 patients prospectively enroled, 47 patients (*n* = 30 female, *n* = 17 male) met inclusion criteria for the study protocol. (Table 1) Of the 22 patients excluded, 16 patients were lost to follow‐up, five patients missed two consecutive tests, and one patient lacked a preoperative thigh circumference measurement. Data on patient characteristics of the excluded patients were not collected. Of the 47 patients studied, mean patient age was 19.1 ± 5.8 years and mean BMI was 23.2 ± 3.3 kg/m^2^. The mean baseline Marx Activity Score was 14.4 ± 2.5. Eighty‐five percent of patients (*n* = 40/47) underwent ACLR using a bone–patellar‐tendon–bone autograft, with the remaining 15% (*n* = 7/47) reconstructed with a hamstring autograft. Meniscal repair was performed in 15 patients (32%), including 10 medial repairs and three lateral repairs, with two patients undergoing medial and lateral meniscal repair. Partial meniscectomy was performed in 12 patients (32%): 1 medial, 10 lateral and 1 medial and lateral.

**Table 1:** Overview of patient participation, mean LSI of thigh circumference, mean LSI of single‐leg hop for distance, mean overall DMA and mean time on the platform.

Testing trial	Patients completing trial (*N*)	Thigh circumference at 10 cm (cm)	Thigh circumference at 20 cm (cm)	Single‐leg hop for distance (cm)	Overall DMA score	Time on platform (s)
Preoperative	47	99.0 ± 2.5	98.3 ± 2.0	NR	596.0 ± 144.4	86.5 ± 15.2
Postoperative						
3 months	44	96.9 ± 3.5	97.0 ± 3.5	NR	500.1 ± 154.4	95.2 ± 16.6
6 months	44	98.0 ± 3.4	98.6 ± 2.5	81.4 ± 13.8	422.9 ± 151.5	102.9 ± 15.0
9 months	41	98.5 ± 2.8	98.2 ± 2.2	87.9 ± 11.3	408.6 ± 151.9	103.9 ± 15.3
12 months	43	98.6 ± 2.7	98.9 ± 2.4	92.9 ± 6.8	371.8 ± 151.6	107.3 ± 14.9
15 months	42	98.8 ± 2.9	98.9 ± 2.3	94.7 ± 7.0	368.1 ± 139.5	107.3 ± 14.1
18 months	38	99.7 ± 2.9	99.2 ± 2.0	95.4 ± 7.0	359.7 ± 152.3	107.5 ± 14.2
24 months	34	NR	NR	NR	315.1 ± 128.9	111.7 ± 12.3

*Note*: Data is presented as mean ± SD.

Abbreviations: cm, centimetres; DMA, dynamic motion analysis; LSI, limb symmetry index; *N*, number of patients; NR, not recorded; SD, standard deviation.

### Thigh circumference

**Figure 1:**
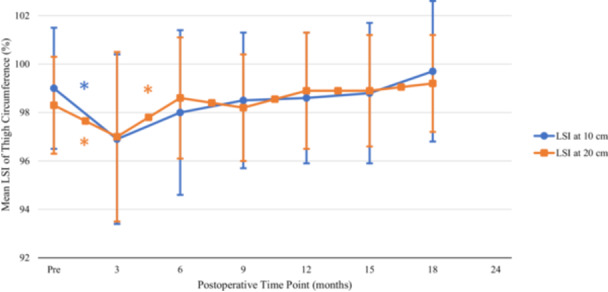
Change in limb symmetry index of thigh circumference. LSI of thigh circumference at 10 cm and 20 cm proximal from the centre of patella over 18 months. Error bars represent SD. *Statistically significant improvement compared with prior LSI of thigh circumference. cm, centimetres; LSI, limb symmetry index; Pre, preoperative; SD, standard deviation, %, percentage.

There was a significant decrease in the LSI for thigh circumference (measured at both 10 and 20 cm proximal to the centre of the patella) at 3‐month follow‐up when compared to preoperative values (Table 1 and Figure 1). There was a significant increase at 20 cm between 3‐ and 6‐month follow‐up.

### Single‐leg hop for distance

**Figure 2:**
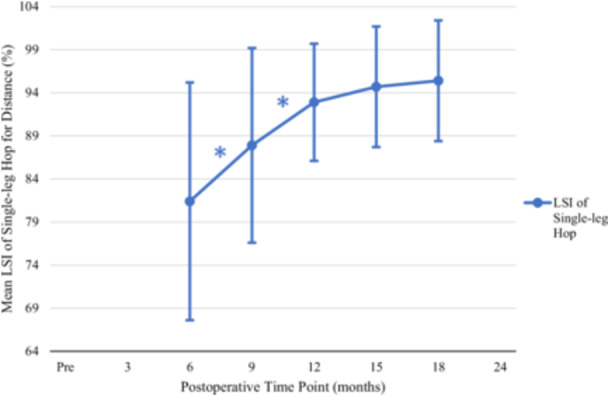
Change in limb symmetry index of single‐leg hop for distance. LSI of single‐leg hop for distance over 18 months. Error bars represent SD. *Statistically significant improvement compared with prior LSI of single‐leg hop for distance. LSI, limb symmetry index; Pre, preoperative; SD, standard deviation; %, percentage.

The LSI for SLHD improved (increased) significantly from the 6‐ to 9‐month and 9‐ to 12‐month time points consecutively (Table 1 and Figure 2). Post hoc power analysis found this study to be sufficiently powered with only a minimum necessary sample size of 19 patients.

### Dynamic postural analysis

**Figure 3:**
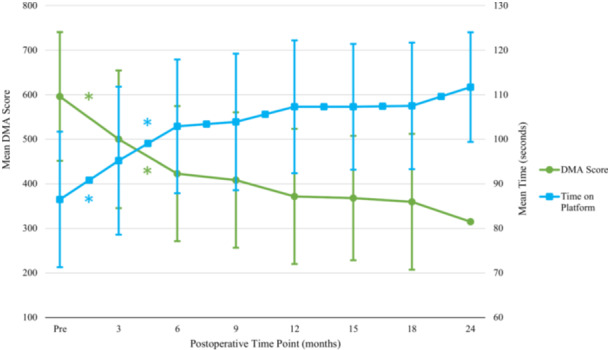
Change in dynamic motion stability testing. Overall dynamic postural stability based on dynamic motion assessment score over 24 months. Error bars represent SD. *Statistically significant improvement compared with prior dynamic motion analysis (DMA). Pre, preoperative; SD, standard deviation.

**Figure 4:**
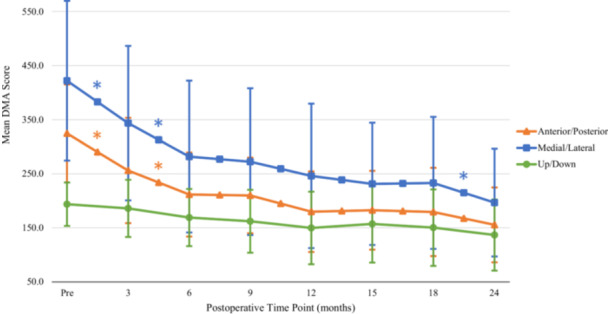
Change of dynamic stability in translational plane. Plane‐specific dynamic stability up to 24 months postoperatively in the translational planes. Error bars represent SD. *Statistically significant improvement compared with prior dynamic motion analysis (DMA) score/time trial. Pre, preoperative; SD, standard deviation.

Overall, mean DMA scores significantly improved (decreased) from the preoperative to 3‐month and 3‐ to 6‐month time points (Table 1 and Figure 3). The length of time that patients remained on the platform significantly improved (increased) from the preoperative‐ to 3‐month and 3‐ to 6‐month time points (Table 1 and Figure 3). Analysis of the mean DMA scores based on individual translational planes of motion demonstrated that stability in the medial/lateral and anterior/posterior planes significantly improved out to 6 months following surgery (Table 1 and Figure 4). There was significant improvement from 18 to 24 months in medial/lateral stability. There were no significant changes in mean DMA scores in the rotational planes of motion. Left/right and internal/external rotational plane measurements remained stable throughout the study period (Table 1). When assessing correlation between LSI of thigh circumference and SLHD on DMA scores (overall and in each individual plane of motion) and time on the platform, no significant association was appreciated at any time point (Appendix A).

When assessing the differences between sex and meniscal treatment preoperatively, females had significantly greater LSI at the 20 cm measurement compared to males. Postoperatively, females had significantly greater LSI than males but only on the 10 cm measurement at the 6‐month and 12‐month time intervals. (Appendices B and C) The only significant relationships found among meniscal surgery groups were lower DMA scores, higher time maintaining feet on the platform, and greater AP translation in patients with no meniscal treatment compared to those who underwent a meniscectomy.

## DISCUSSION

There were several main findings from this study. We found that distal thigh circumference decreased at 3 months, but increased significantly more proximally at 6 months. Single‐leg hop for distance increased from 6 months to 9 months and from 9 months to 12 months postoperatively. Dynamic postural stability improved through 6 months, and the time that patients remained on the platform improved from the preoperative value to the 6‐month time points. No significant correlation was appreciated at any time point between LSI in thigh circumference or SLHD and overall or individual DMA planes of motion, as well as time on the testing platform, at any time point, contradicatory to our hypothesis.

Quadriceps strength has been hypothesised to provide a substantial contibution to lower extremity function following ACLR [25], with postoperative atrophy resulting in altered movement patterns [18, 39], decreased functional performance [17, 27], and an increased risk of re‐injury [15, 33]. In a systematic review, Brown et al. [10] demonstrated that early quadriceps strength deficits can be expected to improve gradually with incremental time intervals (0–6 months, 6–18 months and 18–48 months) from ACL surgery. In our study, during the early (3‐month) postoperative period in which thigh asymmetry was greatest, significant improvements in dynamic postural stability (overall DMA, medial/lateral and anterior/posterior planes) was appreciated, suggesting a disassociation between improvement in dynamic postural stability and restoration of thigh circumference. The lack of correlation between thigh circumference, LSI for SLHD and dynamic postural stability suggests movement patterns that maintain dynamic stability are different than those necessary to propel participants during SLHD testing. As such, there may be significant contributions to dynamic postural stability from core and gluteal strength which are not typically measured objectively in clinical practice or in a research setting. Rodrigues et al. discusses gluteal strength benefitting the uninjured limb [38]. This may impact the performance on the SLHD and subsequently lower the LSI. Future investigations evaluating the relationship between thigh circumference, isokinetic strength and dynamic postural stability are warranted to determine if increased thigh circunference and quadriceps strength over time correspond to normalisation of dynamic postural stability following ACLR. Furthermore, graft type choice may have impacted the results. Cristiani et al. reported a poorer performance in quadriceps strength and SLH testing in patients undering ACLR with BPTB autografts, while poorer hamstrings strength was found in patients receiving HT autograft [11].

Limb symmetry index for SLHD was significantly improved from the 6‐ to 12‐month time intervals, with a trend towards gradual improvement up to 18‐months postoperatively. The SLHD has been used as a gross measurement to quantify the total amount of work performed by an individual's lower extremity kinetic chain during the propulsion phase. Unfortunately, the LSI in SLHD likely fails to capture subtle differences in limb kinematics and kinetic asymmetries [23, 24, 43]. Despite this lack of sensitivity, the LSI in SLHD has been commonly reported as a functional test to assist in determining a patient's ability to return to sports (RTS). Specifically, patients undergoing ACLR have been shown to possess alterations in movement in both the operative and contralateral lower extremity. Goerger et al. [14] observed alterations in movement patterns in both the injured and uninjured lower extremities, both prior to and following ACLR, in 31 participants, potentially increasing the risk for future injury. As such, reliance on LSI for SLHD may overestimate a patient's postural stability during sports and other dynamic activities. Additionally, kinesiophobia postoperatively has been show to impact patient outcomes and RTS [30, 31]. This psychological apprehension may contribute to the poorer LSI SLHD and DMA scores found in this study, especially during the early postoperative period.

Limitations of this investigation include the lack of age, sex or activity‐matched control participants. The mean age was 19 years, which may limit the generalisability of the results in this study to older patients. Despite differences in graft types utilised (bone‐patellar tendon‐bone versus hamstring autograft with allograft augmentation), patients were grouped together as a result of our small sample size. Moreover, the performance of concomitant meniscal procedures (meniscectomy versus repair) were also included, potentially confounding the outcomes and limiting our ability to discern the influence of meniscal injury/treatment on dynamic postural stability measurements following ACLR. We did not separately analyse subgroups by sex or concomitant meniscus injury/treatment since such an analysis would have resulted in an inadequate sample size. Given the superior single‐leg hop distance and hamstring strength of females previously reported in the literature [4], the higher prorportion of females in this study may have impacted the results. Rehabilitation focused on the reacquisition of intrinsic knee sensation is important to restore proprioception and postural stability following ACLR. In this study, patients were provided with a standardised rehabilitation protocol. However, physical therapy was not performed at the same location or by the same therapists, inviting the potential for individual differences in protocol achievement. In addition, we were not able to determine patient compliance with the rehabilitation protocol. This variability in rehabilitation is a limitation but also a potential positive as it makes the study more generalisable. As RTS rate and timing were not recorded, the authors were unable to correlate changes in LSI for thigh circumference, SLHD, and dynamic postural stability to RTS. We recognise that assessing double‐limb rather than single‐limb stability may have some drawbacks. Unfortunately, we did not have equipoise testing of the injured limb in isolation—particularly at early time points—and a significant portion of activities of daily living and sports are performed with both lower extremities. The study may have been underpowered to detect changes at later time points. Future studies of a larger cohort could determine whether the trends toward continued improvement that we noted are significant. Lastly, no *a priori* power analysis was performed to determine appropriate sample size due to the absence of prior data utilising the PROPRIO 5000 for this purpose.

## CONCLUSION

LSI in thigh circumference decreases initially after ACLR and then improves while LSI in SLHD and dynamic postural stability improve after ACLR. Dynamic postural stability was also noted to improve, primarily in translational planes of motion. No significant association between LSI in thigh circumference/SLHD and dynamic postural stability was found at any follow‐up point. These findings suggest parallel but distinct improvements in functional strength and dynamic stability following ACLR.

## AUTHOR CONTRIBUTIONS

**Aguri Kamitani**: Data curation; formal analysis; methodology; validation; writing–original draft; writing–review and editing. **Derrick M. Knapik**: Conceptualisation; investigation; methodology; writing–original draft; writing–review and editing. **Matthew V. Smith**: Investigation; writing–review and editing. **John Motley**: Investigation. **Sina Tartibi**: Data curation; formal analysis; visualisation; writing–review and editing. **Amanda Haas**: Project administration. **Rick Wright**: Investigation; writing–review and editing. **Matthew J. Matava**: Investigation; writing–review and editing. **Robert H. Brophy**: Conceptualisation; investigation; methodology; writing–review and editing.

## CONFLICT OF INTEREST STATEMENT

Derrick M. Knapik: Arthrex Inc. (grant support), Smith & Nephew (education), Elite Orthopaedics (education). Matthew V. Smith: Elite Orthopaedics (speaker, education), Arthrex (consultant), Flexion Therapeutics (Consultant). Rick Wright: Response Arthroscopy LLC (Royalties). Matthew J. Matava: Arthrex Inc. (Educational activities, travel and lodging), Elite Orthopaedics (education), Apollo Orthopaedics (education), Pacira Pharmaceuticals (consultant), Heron Therapeutics (consultant). Robert H. Brophy: Arthrex Inc (speaker, services other than consulting, hospitality payments, education), Sanoft (consultant, hospitality payments), Elite Orthopaedics (education), Smith & Nephew (education), BREGG (education). The other authors declare no conflicts of interest.

## ETHICS STATEMENT

Washington University Institutional Review Board approved this study. Informed consent was obtained, and participants' rights were protected.

## Data Availability

Data available in article supplementary material.

## Appendix A

See Table A1.

**Table A1:** Correlation coefficients between Limb Symmetry Index scores and scores of overall and individual planes of dynamic motion analysis.

	DMA	Time	AP	ML	UD	FE	LR	Rot
Preoperative								
LSI	*R* 0.24	*R* −0.24	*R* 0.13	*R* 0.2	*R* 0.13	*R* −0.21	*R* −0.17	*R* −0.25
10 cm	*p *= 0.11	*p* = 0.10	*p *= 0.382	*p *= 0.18	*p* = 0.38	*p *= 0.17	*p* = 0.25	*p* = 0.095
LSI	*R* −0.12	*R* 0.13	*R* −0.275	*R* −0.0945	*R* −0.15	*R* 0.13	*R* 0.22	*R* 0.17
20 cm	*p *= 0.44	*p *= 0.38	*p *= 0.061	*p *= 0.53	*p *= 0.31	*p *= 0.37	*p *= 0.15	*p* = 0.26
3 month								
LSI	*R* −0.10	*R* 0.14	*R* −0.22	*R* −0.063	*R* −0.28	*R* 0.027	*R* 0.212	*R* 0.164
10 cm	*p* = 0.52	*p* = 0.37	*p* = 0.15	*p* = 0.69	*p *= 0.063	*p *= 0.86	*p* = 0.17	*p* = 0.29
LSI	0.016	*R* 0.033	*R* −0.11	*R* 0.055	*R* −0.3	*R* −0.08	*R* 0.15	*R* 0.096
20 cm	*p *= 0.92	*p* = 0.83	*p* = 0.50	*p *= 0.72	*p *= 0.051	*p *= 0.61	*p *= 0.33	*p *= 0.54
6 month								
LSI	*R* −0.22	*R* 0.19	*R* −0.13	*R* −0.19	*R* −0.24	*R* 0.01	*R* −0.051	*R* 0.04
10 cm	*p* = 0.25	*p *= 0.32	*p *= 0.50	*p *= 0.30	*p *= 0.21	*p *= 0.96	*p* = 0.79	*p *= 0.83
LSI	*R* −0.31	*R* 0.29	*R* −0.30	*R* −0.27	*R* −0.33	*R* 0.118	*R* 0.132	*R* 0.27
20 cm	*p* = 0.085	*p *= 0.12	*p* = 0.10	*p* = 0.15	*p* = 0.077	*p* = 0.54	*p *= 0.49	*p *= 0.16
LSI	*R* −0.17	*R* 0.11	*R* −0.11	*R* −0.17	*R* −0.17	*R* −0.01	*R* 0.043	*R* −0.0002
SLHD	*p* = 0.35	*p *= 0.56	*p *= 0.58	*p* = 0.38	*p *= 0.38	*p* = 0.96	*p *= 0.82	*p *= 1
9 month								
LSI	*R* −0.093	*R* 0.13	*R* −0.17	*R* −0.050	*R* −0.17	*R* 0.126	*R* 0.20	*R* 0.16
10 cm	*p* = 0.56	*p *= 0.44	*p* = 0.30	*p *= 0.76	*p* = 0.30	*p* = 0.43	*p* = 0.21	*p* = 0.32
LSI	*R* −0.29	*R* 0.27	*R* −0.21	*R* −0.23	*R* −0.32	*R* 0.096	*R* 0.142	*R* 0.115
20 cm	*p* = 0.072	*p* = 0.092	*p* = 0.18	*p *= 0.15	*p *= 0.043	*p *= 0.55	*p* = 0.38	*p *= 0.47
LSI	*R* −0.14	*R* 0.12	*R* −0.071	*R* −0.12	*R* −0.14	*R* 0.0011	*R* 0.11	*R* 0.10
SLHD	*p* = 0.37	*p *= 0.51	*p *= 0.66	*p* = 0.47	*p* = 0.39	*p *= 0.99	*p *= 0.49	*p *= 0.53
12 month								
LSI	*R* −0.09	*R* 0.079	*R* −0.19	*R* 0.008	*R* −0.24	*R* −0.14	*R* −0.07	*R* −0.11
10 cm	*p* = 0.60	*p* = 0.68	*p* = 0.29	*p *= 0.97	*p* = 0.21	*p* = 0.45	*p *= 0.69	*p *= 0.57
LSI	*R* −0.21	*R* 0.21	*R* −0.28	*R* −0.17	*R* −0.16	*R* 0.16	*R* 0.113	*R* 0.083
20 cm	*p* = 0.26	*p* = 0.27	*p *= 0.14	*p *= 0.40	*p* = 0.40	*p* = 0.39	*p *= 0.55	*p *= 0.66
LSI	*R* −0.12	*R* 0.062	*R* −0.050	*R* −0.13	*R* −0.072	*R* −0.21	*R* −0.16	*R* −0.060
SLHD	*p* = 0.52	*p* = 0.75	*p* = 0.78	*p* = 0.49	*p* = 0.71	*p* = 0.27	*p *= 0.40	*p *= 0.76
15 month								
LSI	*R* −0.091	*R* 0.061	*R* −0.063	*R* −0.12	*R* −0.20	*R* −0.099	*R* 0.077	*R* 0.057
10 cm	*p* = 0.57	*p* = 0.70	*p *= 0.69	*p* = 0.43	*p* = 0.20	*p *= 0.53	*p* = 0.62	*p *= 0.72
LSI	*R* −0.23	*R* 0.21	*R* −0.16	*R* −0.26	*R* −0.27	R 0.11	*R* 0.088	*R* 0.12
20 cm	*p* = 0.14	*p* = 0.18	*p* = 0.32	*p* = 0.099	*p* = 0.084	*p* = 0.50	*p *= 0.58	*p* = 0.45
LSI	*R* −0.055	*R* 0.068	*R* −0.060	*R* −0.11	*R* −0.071	*R* −0.019	*R* 0.036	*R* −0.0085
SLHD	*p* = 0.73	*p *= 0.67	*p* = 0.70	*p* = 0.50	*p* = 0.652	*p* = 0.91	*p *= 0.82	*p* = 0.96
18 month								
LSI	*R* −0.24	*R* 0.088	*R* 0.16	*R* −0.25	*R* −0.15	*R* −0.06	R −0.25	*R* −0.25
10 cm	*p* = 0.2	*p* = 0.64	*p* = 0.39	*p* = 0.18	*p* = 0.43	*p* = 0.72	*p* = 0.18	*p* = 0.18
LSI	*R* −0.20	*R* 0.13	*R* −0.12	*R* −0.08	*R* −0.16	*R* 0.18	*R* −0.10	*R* −0.005
20 cm	*p* = 0.28	*p *= 0.48	*p* = 0.52	*p* = 0.65	*p *= 0.40	*p *= 0.35	*p* = 0.59	*p* = 0.98
LSI	*R* −0.32	*R* 0.24	*R* −0.28	*R* −0.29	*R* −0.27	*R* 0.055	*R* −0.12	*R* −0.009
SLHD	*p* = 0.089	*p* = 0.20	*p* = 0.13	*p* = 0.12	*p *= 0.15	*p *= 0.77	*p *= 0.53	*p* = 0.96

Abbreviations: AP, anterior/posterior; cm, centimetres; DMA, overall dynamic motion analysis; FE, flexion/extension; LR, left/right; LSI, limb symmetry index; LSI10cm, LSI of thigh girth measured at 10 cm proximal from the centre of the patella; LSI20cm; LSI of thigh circumference measured at 20 cm proximal from the centre of patella; LSI SLHD; LSI of single‐leg hop for distance; ML, medial/lateral; *p*, *p*‐value; *R*, correlation coefficient; Rot, internal/external rotation; UD, up/down.

## Appendix B

See Table B1.

**Table B1:** Sex based comparison of clinical measurements.

	LSI 10 cm	LSI 20 cm	LSI SLHD	DMA	Time	AP	ML	UD	FE	LR	Rot
Preoperative (female, *n* = 30; male, *n* = 17)											
Female	99.4 ± 2.7	98.9 ± 1.8		620.6 ± 162.1	84.3 ± 17.1	328.7 ± 101.5	448.8 ± 161.8	189.7 ± 40.2	417.7 ± 163.6	364.1 ± 171.8	459.6 ± 207.5
Male	98.3 ± 2.2	97.3 ± 2.1		552.6 ± 102.5	90.3 ± 11.3	317.3 ± 69.1	374.6 ± 107.0	200.8 ± 40.4	441.3 ± 138.8	322.2 ± 94.9	435.2 ± 137.0
*p*‐Value	0.17	0.02*		0.13	0.21	0.69	0.10	0.37	0.62	0.36	0.67
3 month (female, *n* = 27; male, *n* = 17)											
Female	97.5 ± 3.9	97.4 ± 3.9		504.7 ± 168.0	95.4 ± 18.0	252.7 ± 106.0	355.0 ± 156.4	181.5 ± 55.6	499.2 ± 170.9	382.0 ± 161.6	514.0 ± 200.4
Male	95.9 ± 2.7	96.4 ± 2.7		492.9 ± 140.1	95.2 ± 15.2	261.4 ± 84.8	325.6 ± 120.5	193.2 ± 49.1	476.7 ± 167.3	316.2 ± 137.7	439.0 ± 169.3
*p*‐Value	0.15	0.37		0.81	0.97	0.78	0.51	0.48	0.67	0.17	0.21
6 month (female, *n* = 27; male, *n* = 17)											
Female	98.9 ± 3.8	99.2 ± 2.7	80.3 ± 13.6	408.7 ± 158.1	104.4 ± 15.6	201.7 ± 79.6	275.4 ± 148.0	159.0 ± 51.6	551.9 ± 151.1	372.8 ± 109.7	516.2 ± 140.6
Male	96.6 ± 2.3	97.7 ± 2.0	83.1 ± 14.8	445.5 ± 147.0	100.6 ± 14.5	227.4 ± 74.2	292.0 ± 131.3	184.9 ± 52.5	499.2 ± 131.6	350.0 ± 136.3	481.6 ± 183.2
*p*‐Value	0.034*	0.06	0.52	0.44	0.43	0.29	0.71	0.11	0.24	0.55	0.48
9 month (female, *n* = 27; male, *n* = 14)											
Female	99.0 ± 2.8	98.4 ± 2.1	86.0 ± 12.2	399.7 ± 172.3	104.5 ± 16.8	290.8 ± 504.5	276.0 ± 158.7	148.9 ± 54.2	532.1 ± 172.1	374.7 ± 138.1	489.4 ± 183.5
Male	97.4 ± 2.6	98.1 ± 2.1	91.5 ± 9.3	425.8 ± 113.7	102.7 ± 13.0	226.0 ± 77.7	265.4 ± 78.8	187.8 ± 59.1	504.5 ± 147.4	346.7 ± 185.6	461.3 ± 178.5
*p*‐Value	0.08	0.63	0.15	0.6	0.73	0.64	0.82	0.41	0.61	0.59	0.64
12 month (female, *n* = 28; male, *n* = 14)											
Female	99.3 ± 2.5	99.2 ± 2.6	92.3 ± 7.1	353.2 ± 153.3	109.0 ± 15.0	168.4 ± 72.7	237.8 ± 137.8	135.4 ± 53.7	557.1 ± 150.2	399.5 ± 115.2	526.2 ± 150.9
Male	97.0 ± 2.7	98.4 ± 1.9	94.2 ± 6.5	409.1 ± 152.3	103.8 ± 15.3	202.9 ± 75.6	262.6 ± 128.0	178.8 ± 82.7	517.4 ± 148.9	384.0 ± 206.8	474.2 ± 148.1
*p*‐Value	0.01*	0.34	0.39	0.27	0.30	0.16	0.58	0.05	0.42	0.76	0.30
15 month (female, *n* = 26; male, *n* = 16)											
Female	99.1 ± 3.2	98.9 ± 2.6	93.6 ± 7.4	350.9 ± 144.0	108.5 ± 14.4	174.1 ± 73.0	220.7 ± 119.4	147.4 ± 65.6	532.0 ± 131.6	346.0 ± 103.3	478.3 ± 129
Male	98.2 ± 2.4	98.9 ± 2.0	96.5 ± 6.3	395.9 ± 136.6	105.5 ± 14.4	196.2 ± 73.0	248.5 ± 103.2	173.1 ± 79.1	523.2 ± 162.1	360.8 ± 156.9	461.9 ± 166.5
*p*‐Value	0.33	0.93	0.21	0.32	0.53	0.35	0.45	0.26	0.85	0.71	0.72
18 month (female, *n* = 23; male, *n* = 14)											
Female	99.7 ± 2.6	99.4 ± 2.0	94.2 ± 6.8	339.5 ± 162.8	109.1 ± 15.0	169.5 ± 83.5	222.1 ± 129.7	137.2 ± 70.2	529.0 ± 133.9	345.7 ± 96.0	467.4 ± 110.5
Male	99.7 ± 3.5	98.7 ± 2.1	97.3 ± 7.4	392.8 ± 139.0	104.9 ± 13.5	195.6 ± 79.0	251.1 ± 110.5	172.2 ± 68.4	503.8 ± 137.5	339.0 ± 144.0	420.4 ± 139.5
*p*‐Value	0.98	0.29	0.22	0.32	0.4	0.35	0.49	0.15	0.57	0.87	0.26
24 month (female, *n* = 21; male, *n* = 13)											
Female				305.5 ± 130.6	112.9 ± 12.2	146.8 ± 66.0	194.2 ± 105.6	127.9 ± 58.8	569.6 ± 138.0	361.5 ± 97.3	509.1 ± 140.0
Male				330.6 ± 134.9	109.7 ± 13.2	169.7 ± 74.4	200.7 ± 93.4	151.2 ± 75.8	536.7 ± 130.5	314.4 ± 75.8	445.6 ± 117.1
*p*‐Value				0.6	0.49	0.36	0.86	0.32	0.50	0.15	0.18

Abbreviations: AP, anterior/posterior; cm, centimetres; DMA, overall dynamic motion analysis; FE, flexion/extension; LR, left/right; LSI, limb symmetry index; LSI10cm, LSI of thigh girth measured at 10 cm proximal from the centre of the patella; LSI20cm; LSI of thigh circumference measured at 20 cm proximal from the centre of patella; LSI SLHD; LSI of single‐leg hop for distance; ML, medial/lateral; *p*, *p*‐value; Rot, internal/external rotation; UD, up/down.

* Statistically significant difference between males and females. Clinical variables were reported as mean ± standard deviation.

## Appendix C

Table C1

**Table C1:** *p*‐Value in concomitant meniscal treatment‐based comparison of clinical measurements.

	LSI 10 cm	LSI 20 cm	LSI SLHD	DMA	Time	AP	ML	UD	FE	LR	Rot
Preoperative (*N*, *n* = 10; M, *n *= 12; R, *n* = 15)											
N‐M	0.47	1.00		0.13	0.12	0.2	0.27	1.00	0.13	0.74	1.00
M‐R	0.92	1.00		0.29	0.45	1.00	0.38	1.00	0.69	0.58	1.00
R‐N	0.69	0.74		1.00	1.00	1.00	1.00	1.00	1.00	0.15	1.00
3 month (N, *n* = 19; M, *n* = 10; R, *n* = 15)											
N‐M	1.00	1.00		0.1	0.19	0.16	0.16	1.00	0.67	1.00	1.00
M‐R	0.98	1.00		1.00	1.00	1.00	1.00	1.00	1.00	0.48	1.00
R‐N	0.94	1.00		1.00	1.00	0.38	1.00	1.00	0.86	0.83	1.00
6 month (N, *n* = 20; M, *n* = 10; R, *n *= 14)											
N‐M	1.00	1.00	0.92	0.14	0.22	0.12	0.16	0.54	0.99	1.00	1.00
M‐R	1.00	1.00	0.37	1.00	1.00	1.00	0.94	1.00	1.00	0.12	1.00
R‐N	1.00	1.00	1.00	0.76	0.80	0.53	0.96	1.00	1.00	0.36	1.00
9 month (N, *n* = 19; M, *n* = 9; R, *n* = 13)											
N‐M	0.34	1.00	1.00	0.05	0.06	0.05	0.08	0.21	0.11	1.00	0.94
M‐R	1.00	0.65	1.00	1.00	1.00	1.00	1.00	1.00	1.00	0.07	0.35
R‐N	0.20	0.93	1.00	1.00	1.00	1.00	1.00	1.00	1.00	0.39	1.00
12 month (N, *n* = 21; M, *n* = 9; R, *n* = 13)											
N‐M	1.00	1.00	1.00	0.79	1.00	0.71	0.94	0.6	1.00	1.00	1.00
M‐R	0.13	1.00	1.00	1.00	1.00	1.00	1.00	1.00	1.00	0.46	1.00
R‐N	0.17	1.00	1.00	1.00	1.00	1.00	1.00	1.00	1.00	0.55	1.00
15 month (N, *n* = 21; M, *n* = 7; R, *n* = 14)											
N‐M	1.00	0.38	1.00	0.26	0.53	0.33	0.44	0.21	1.00	1.00	1.00
M‐R	1.00	0.58	0.33	0.73	0.46	0.86	0.96	0.47	0.59	1.00	0.20
R‐N	0.37	0.47	0.033*	1.00	1.00	1.00	1.00	0.50	1.00	1.00	0.23
18 month (N, *n* = 19; M, *n* = 8; R, *n* = 11)											
N‐M	1.00	1.00	0.46	0.54	1.00	1.00	0.50	0.8	1.00	1.00	0.91
M‐R	0.19	0.91	0.39	1.00	1.00	1.00	0.94	1.00	1.00	0.07	0.22
R‐N	0.50	0.15	1.00	1.00	1.00	1.00	1.00	1.00	1.00	0.9	1.00
24 month (N, *n* = 15; M, *n* = 8; R, *n* = 11)											
N‐M				0.49	0.67	0.85	0.36	1.00	1.00	1.00	1.00
M‐R				1.00	0.75	0.60	1.00	1.00	0.09	0.29	1.00
R‐N				1.00	1.00	1.00	1.00	1.00	1.00	0.95	1.00

Abbreviations: AP, anterior/posterior; cm, centimetres; DMA, overall dynamic motion analysis; FE, flexion/extension; LR, left/right; LSI, limb symmetry index; LSI10cm, LSI of thigh girth measured at 10 cm proximal from the centre of the patella; LSI20cm; LSI of thigh circumference measured at 20 cm proximal from the centre of patella; LSI SLHD; LSI of single‐leg hop for distance; M, meniscectomy; ML, medial/lateral; N, no meniscal treatments; *p*, *p*‐value; R. meniscal repair; Rot, internal/external rotation; UD, up/down.

* Statistically significant difference among meniscal treatments.
